# The Fifth German Oral Health Study (Fünfte Deutsche Mundgesundheitsstudie, DMS V) – rationale, design, and methods

**DOI:** 10.1186/1472-6831-14-161

**Published:** 2014-12-29

**Authors:** Rainer A Jordan, Constanze Bodechtel, Katrin Hertrampf, Thomas Hoffmann, Thomas Kocher, Ina Nitschke, Ulrich Schiffner, Helmut Stark, Stefan Zimmer, Wolfgang Micheelis, the DMS V Surveillance Investigators’ Group

**Affiliations:** Institute of German Dentists (IDZ), Universitaetsstrasse 73, 50931 Cologne, Germany; Kantar Health GmbH, Munich, Germany; Clinic of Oral and Maxillofacial Surgery, Faculty of Medicine, Christian Albrechts University, Kiel, Germany; Department of Periodontology, Faculty of Medicine, Carl Gustav Carus, Technische Universität Dresden, Dresden, Germany; Unit of Periodontology, School of Dentistry, Ernst Moritz Arndt University Medicine, Greifswald, Germany; Clinic for Geriatric and Special Care Dentistry, Center of Dental Medicine, University of Zurich, Zurich, Switzerland; Department of Restorative and Preventive Dentistry, Center for Dental and Oral Medicine, University Medical Center Hamburg-Eppendorf, Hamburg, Germany; Department of Prosthodontics, Preclinical Education, and Dental Materials Science, Rhenish Friedrich Wilhemls University of Bonn, Bonn, Germany; Department of Operative and Preventive Dentistry, School of Dental Medicine, Faculty of Health, Witten/Herdecke University, Witten, Germany

**Keywords:** Behavioral research, Cross-sectional studies, Dental caries, Dentistry, Epidemiology, Health care surveys, Oral health, Periodontal diseases, Prosthodontics, Sense of coherence, Social class, Social science

## Abstract

**Background:**

Oral diseases rank among the most prevalent non-communicable diseases in modern societies. In Germany, oral epidemiological data show that both dental caries and periodontal diseases are highly prevalent, though significant improvements in oral health has been taking in the population within the last decades, particularly in children. It is, therefore, the aim of the Fifth German Oral Health Study (DMS V) to actualize the data on current oral health status and to gather information on oral health behavior and risk factors. In addition to current oral health monitoring, the study will also permit conclusions about trends in the development of oral health in Germany between 1989 and 2014.

**Methods/Design:**

DMS V is a cross-sectional, multi-center, nationwide representative, socio-epidemiological study to investigate the oral health status und behavior of the German resident population in four age cohorts. Study participants are children (12-year-olds), adults (35- to 44-year-olds), young olds (65- to 74-year-olds), and old olds (75- to 100-year-olds) who are drawn from local residents’ registration offices. Social-science investigation parameters concern subjective perceptions and attitudes regarding oral health and nutrition, sense of coherence, and socio-demographic data. Clinical oral parameters are tooth loss, caries and periodontitis, prosthodontic status, further developmental and acquired dental hard tissue and mucosal lesions. To ensure reproducibility, the dental investigators are trained and calibrated by experts and multiple reliability checks are performed throughout the field phase. Statistical analyses are calculated according to a detailed statistical analysis plan.

**Discussion:**

The DMS studies first performed in 1989, 1992 and repeated in 1997 and 2005 are the only cross-sectional oral health studies conducted in Germany on a population-based national representative level. Updated prevalence and trend analyses of key oral diseases are, therefore, of major epidemiological and health services research interest.

**Trial registration:**

German Health Services Research Data Bank VfD_DMSV_13_002152

## Background

Oral diseases rank among the most prevalent non-communicable diseases in modern societies and require a substantial due of health care systems. In Germany, epidemiological data concerning the prevalence of oral diseases show that both dental caries and periodontal diseases are highly prevalent. Basically, Germany shares this situation with the other European and non-European developed nations [1, 2]. However, when comparing prevalence data over time, it becomes apparent that in Germany significant improvements in oral health can be detected in the population, particularly in children [3–5]. In children, Germany has been joining those countries being at the top of international oral health since years [6]. Caries experience in the Western German federal states as expressed by DMFT declined from 6.8 in the 1980s to 0.7 in 2005 [4, 7]. In the Eastern German federal states, respective epidemiologic data declined from 3.4 to 1.1 [8], also demonstrating that still differences in oral health between old West German states and newly-formed German states exist [5]. With regard to adult oral health, a clear increase in natural tooth preservation can be ascertained within the last decades [9]. On the other hand, these oral health developments have the consequence that the treatment burden of periodontal disease and of root caries may increase because more teeth are retained in seniors and aged persons [10]. For the population group beyond age 75 years, so called old olds, there is only very scattered information about oral morbidity from regional studies [11]. However, the available data indicate a high dental disease burden and a low level of health care provision for old olds and care-dependent persons.

As a monitoring instrument, the Institute of German Dentists started to collect epidemiological data on oral health in Germany in 1989 in West Germany (DMS I) [12], followed by a supplementary survey in the newly-formed German states in 1992 after reunification (DMS II) [13]. The Third German Oral Health Study (DMS III) was conducted in 1997 [14], and the fourth (DMS IV) in 2005 [15]. The Fifth German Oral Health Study (DMS V) is now designed as a cross-sectional, multi-center, nationwide representative, socio-epidemiological study in four selected age cohorts. Its purpose is to ascertain the current clinical dental state of oral health using a clinical examination, and to gather information of oral health behavior using a socio-scientific survey at the same time. In addition the study will also allow trend analyses of oral health in Germany between 1989 and 2014.

## Methods

The Fifth German Oral Health Study (DMS V) is a cross-sectional, multi-center, nationwide representative, socio-epidemiological study to investigate the oral health status and behavior of the German resident population in four age cohorts. Study participants are children (12-year-olds), adults (35- to 44-year-olds), young olds (65- to 74-year-olds), and old olds (75- to 100-year-olds) that are randomly drawn from local residents’ registration offices. Social-science investigation parameters are socio-demographic data, subjective perceptions and attitudes regarding oral health, nutrition and sense of coherence (SOC). Clinical dental parameters are caries, periodontitis, prosthodontic status, further developmental and acquired dental hard tissue, and mucosal lesions.

### Study design development

The DMS V study design was developed from 2013 to 2014 by the Institute of German Dentists (IDZ). Main conditions of the survey are performed as in previous DMS studies. The field time and corresponding aspects of the study are planned in collaboration with the operation center in Munich (Kantar Health GmbH), selected after a pan-European call for tender. The operation center is ISO 2052 certified. The clinical dental examination program is developed according to contemporary epidemiological standards with an expert advisory board of seven university professors. The study related hygiene concept was consented with the Robert Koch Institute, Berlin. The study protocol was developed according to the SPIRIT statement recommendations [16].

### Ethics and dissemination

DMS V is approved by the Institutional Review Board (IRB) of the North Rhine medical association, Düsseldorf (DMS V registration number 2013384). The study is further registered at the German Health Services Research Data Bank (DMS V registration number VfD_DMSV_13_002152). Announcements to DMS V are made in local and federal German dental journals to sensitize dentists for the study, as well as articles are published in local newspapers to encourage the public for support. Finally, local mayor’s office, police departments, and other local regulatory authorities are informed about the study.

### Study setting

In accordance with international requirements, the study focuses on selected age strata. As in previous DMS studies, these are 12-year-olds (children), 35- to 44-year-olds (adults) and 65- to 74-year-olds (young olds). In addition, 75- to 100-year-olds (old olds) are sampled for the first time. For the investigations at the 90 study sample points, four teams work in parallel; each includes one dentist, one interviewer, and one contact person. In addition there is also a back-up team. One week before opening the investigation center at the study sample point, the contact person begins to establish contact with all persons who have not yet answered to the operation center despite multiple invitations. Before opening the investigation center, the contact person passes all documents to the interviewer for post-processing. The interviewer is responsible for welcoming the study participants, informing them about data protection, obtaining their informed consent, obtaining responses to the socio-scientific questionnaires and carrying out further follow-up work with the subjects, whereas the dentist performs the clinical assessment.

### Examiner calibration and reliability tests

To ensure that the dental assessments are of high quality, the dental investigators are trained and calibrated by experts. Reliability test are performed three times during the field phase. The reliability tests are carried out for the following target diseases: determination of diseases of the oral mucosa, dental caries assessment, dental erosions, provision of dental prostheses, and measurement of probing depth and recessions. These occur in each phase according to the following model:

1. Children: A total of six test persons aged 12 years are required. In this age cohort, only dental caries and dental erosions are assessed. At the beginning of the reliability test, the dental investigators and the associated expert are distributed across the examination rooms and assess the particular test person. Subsequently, the investigators go one examination room further on and repeat the procedure for the next person encountered etc. Thus, a rotating assessment procedure is performed, until such time as each investigator has investigated the test person she/he first investigated a second time. After this, the reliability test is finished. As a time allocation of ten minutes per investigation for reliability testing in children is envisaged, this process, with six investigators and including a double assessment, requires 70 minutes.
2. Adults/Olds: A similar procedure is followed for adults/olds (age of the test persons 35 and upwards). However, owing to the increased assessment spectrum, reliability tests in this age cohort are divided into two parts. In the first reliability part, the diseases of the oral mucosa and dental caries are checked with five dentists and two experts. Consequently, seven test persons and seven examination rooms are required. In the second reliability part, ultimately the periodontal pocket probing depth, recession and provision with dental prostheses are recorded. Again, seven test persons and treatment rooms are required. In each case, 15 minutes are taken into account for these investigations, so that 105 minutes are calculated for each part of the reliability tests.

The advantage of this calibration model is that both intra-examiner reliability calculations and inter-examiner reliability calculations can be carried out, the latter both between the expert and the dental investigator as well as between the dental investigators. The reliability tests will then be continued on two further occasions during the field phase (in the middle and to the end) according to this model. During the field phase, monitoring takes place by members of the operation center, the principal investigators, and the expert advisory board.

### Timeline

The field phase is scheduled between October 2013 and July 2014. Each team processes one study sample point per week on a total of six working days. In each case, after three weeks of working time, there is a week’s break. All four age cohorts are invited to the investigation center, with the stipulated appointments being on the first three days of the investigation week. The last two days of the week is reserved for investigations through home visits, as it is expected that there will be increased immobility in the cohort of old olds, which in some circumstances makes visiting an investigation center impossible. Likewise, home visits are carried out for all subjects who have basic mobility problems. On days four and five of the week, additional subjects of all age cohorts may be visited who until then saw no possibility of coming to the investigation center. Since the teams only spend a limited time period at one location meaning that some target persons will have problems from a time point of view being able to take part in the investigation at precisely this time, a further follow-up work phase is added on to the regular field phase for ten days.

### Sampling procedure and recruitment

**Figure 1:**
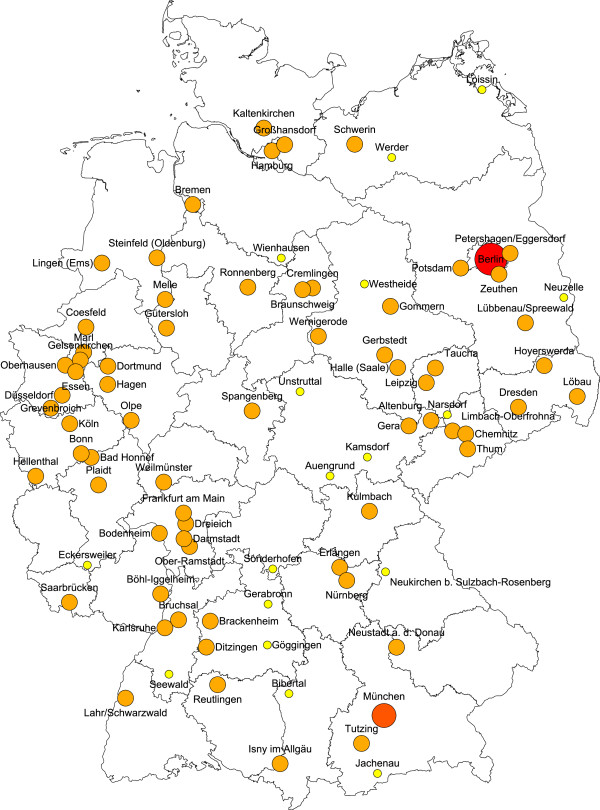
DMS V study sample points*. *Orange marked communities or cities are regular study sample points; in red marked cities, more than 1 study sample point was selected; in yellow marked study sample points, the communities were too small by population to achieve the target number of study participants and a so called synthetic study sample point was built by pooling several surrounding communities.

The investigations are to take place at 90 study sample points, selected randomly to be representative nationwide. For reasons of comparison, a disproportional sample point selection with 60 study sample points in West Germany and 30 study sample points in East Germany (oversampling) is chosen (Figure 1). The basis for selection is a stratification of Germany using the criteria of federal states and the levels of urbanization. The names and addresses of the study participants to be invited (target persons) are drawn from the registration files of the local residents’ registration offices. In each age cohort, it is the aim to include 1,000 subjects (net) into the study. Therefore, 2,000 target persons are drawn from the registration offices in the children, adult, and young olds age cohorts. Due to several reasons of potential reduced accessibility in old olds, 3,000 target persons are drawn for this age cohort from the registration offices. The randomly selected target persons receive letters of invitation with suggested dates for visiting an investigation center, where the socio-scientific survey and the dental examination are carried out. The structure of the letter was pretested with a qualitative study design (paraphrasing of the main essentials of the letter) on a sample of n = 24 persons with different sex, ages, and education levels. Old olds target persons are also offered the alternative of a home visit for examination. In addition, the operation center maintains a free telephone hotline in the event of any questions on the study or for the arrangement of individual appointments. For each of the target persons, an address log is kept which records all the contacts and does so throughout all the field phase. The results of the efforts are given (failure) codes. In this way, the current status can always be reproduced. If it has not been possible to carry out an investigation and no refusal was stated in advance, the target persons are sent a short written questionnaire with key questions as part of a non-response analysis. The analysis of these questionnaires makes it possible to assess, whether distortions of the study results are to be expected through the non-participation of this group of people. All study participants receive a monetary incentive; travel fares will also be reimbursed in individual cases on request.

### Investigation centers

The examinations and surveys are carried out in suitable investigation rooms, generally provided or rented out from local authorities. Since the examination in the field is not carried out under practice conditions, all precautions are taken to ensure the quality of the examination as far as possible. The investigation centers are set up in accordance with the requirements of the dental examination. A simplified mobile dental examination chair (AGA, Löhne, Germany) is available for the clinical examination. Using this chair, it is possible to place the subjects in a half lying position. The oral cavity is illuminated using a halogen lamp with 40,000 lux (Medical Econet, Oberhausen, Germany). Since there is no suction, saliva is absorbed with cotton rolls, if necessary. Periodontal examinations take place at the end of the clinical examination to avoid that provoked bleeding obscures the viewing field. The oral findings are stored with the DentaSoft V software program, which was developed especially for this purpose. Dental instruments used for each person examined are disinfected with a disinfection solution, manually cleansed afterwards, again disinfected with hot air, and stored in closed metal instrument containers. The instructions for the preparation of dental instruments (medical devices) are based on the current recommendations of the Robert Koch Institute [17]. Quality controls of the medical devices disinfection procedures are processed by independent laboratory measurements of instrumental bio-burden at regularly intervals throughout the entire field phase.

### Study end points

#### Social science study end points

A paper questionnaire is completed by the subjects at the investigation centre before clinical examination. The questionnaires were also used from previous DMS studies to ensure comparisons. Further, the questionnaires are designed according to the specific age cohorts. The interviewers provide the subjects with support with this as appropriate. The basic points of the social science survey component with general question parameters are: general perception of oral health, self-efficacy regarding oral health, cognitive attitude regarding monitoring of dental health, snacks between meals, oral hygiene habits/prosthesis hygiene, past periodontal treatment and regular supportive periodontal care, utilization of dental services, loyalty to dentist, subjective satisfaction with dental prosthesis, subjective morbidity status, questions on childhood and course of life, care status, wearing behavior of removable prosthesis, tobacco/alcohol consumption, social demographics, place of residence, and place of birth (if Germany, federal state; otherwise country). For the first time, the sense of coherence scale (SOC-13 [18]) is incorporated to obtain information between health, stress, and coping. The SOC scale consists of three dimensions: comprehensibility, manageability, and meaningfulness.

Particular issues arise among the old olds. As well as being significantly shortened, the foci of the questions for this age group are specific perception of oral health, general perception of health, utilization pattern/care pattern of dental services, subjective satisfaction with dental prosthesis, utilization pattern/care pattern of medical services, perceptions of oral pain, disabled status and degree of disability, care status and care context, any assistance needs for housekeeping, wearing behavior of removable prosthesis, survey on age-specific diet and food intake, general mobility status, reduced social demographics, place of residence, and place of birth (if Germany, then federal state; otherwise country).

#### Clinical study end points

Clinical examinations are applied to the subjects in different age cohorts according to Table 1. The course of clinical examinations is carried out according to the following order:

**Table 1:** **DMS V clinical examinations according to age cohorts**

Clinical study end point	Children (12-year-olds)	Adults (35- to 44-year-olds)	Young olds (65- to 74-year-olds)	Old olds (75- to 100-year-olds)
Oral mucosa lesions		X	X	X
Tooth-specific findings	X	X	X	X
Tooth surface-specific findings	X	X	X	X
Molar incisor hypomineralization	X			
Dental erosions	X	X	X	X
Dental caries	X	X	X	X
Root caries		X	X	X
Periodontal index teeth recording		X (90%)	X (90%)	
Periodontal full-mouth recording		X (10%)	X (10%)	X
Gingivitis	X			
Prosthodontics		X	X	X
Oral functional capability				X

##### Oral mucosa lesions

Examination of the oral mucosa is carried out with two dental mirrors in all subjects ≥ 35 years. Partial dentures are removed if present. The following forms of oral lesions are recorded: carcinoma, leukoplakia, erythroplakia, lichen planus, candida, smoker’s keratosis, prosthesis-related changes, other changes. The lesion size is not recorded. Selection of oral mucosa lesions is based on the recommendations of the WHO [19, 20]. The localization of the lesions is captured using a coding model based on Roed-Petersen and Renstrup [21]. Where findings are present, a photograph of the lesion is taken for a systematic expert diagnostic verification.

##### Tooth-specific findings

Tooth-specific findings are obtained for all teeth including third molars. At the beginning of the examination, each subject is asked whether she/he has a removable prosthesis or any implants. The following findings are recorded: extracted teeth due to caries, not replaced; missing, non-replaced teeth, missing for reasons other than caries; full crown; partial crown (at least one cusp covered); anchor crown (bridge anchor, telescopic crown, crown anchored to bar, root caps); pontic (fixed prosthesis); replaced tooth (removable prosthesis); implant (with prosthetic restoration).

##### Tooth surface-specific findings

In the surface-specific examination, an assessment is performed of five surfaces per posterior tooth (premolars and molars) and four surfaces per anterior tooth (incisors and canines). An assessment is performed in teeth, which have been erupted into the oral cavity at least beyond the equator. The following findings are recorded on surface level: initial caries (for details see below); carious lesions (for details see below); secondary caries (for details see below); fissure sealants; dental restorations (the restoration material is not recorded).

##### Molar incisor hypomineralization

Special interest shall be put on recording teeth showing signs of molar incisor hypomineralization (MIH). This kind of developmental tooth defect with hitherto unknown aetiology is by definition restricted to incisors and molars, although other teeth can show the same characteristics. For documentation, the MIH index according to the European Academy of Paediatric Dentistry is used [22]. Each tooth is assessed using the MIH code definitions: Code 1: limited demarcated opacities; mildest form of MIH, no surface loss; generally, whitish or yellowish, occasionally also brownish discolored areas can be identified as a result of the disordered mineralization. Opacities smaller than 1 mm are not recorded. Code 2: posteruptive, localized enamel cracks. Code 3: posteruptive enamel breakdown, large scale. Code 4: atypical restorations. Code 5: extraction due to MIH. Code 6: tooth has not erupted.

##### Dental erosions

Erosions are measured according to the basic erosive wear examination (BEWE) [23]. The most severe finding of a sextant is registered. Typical for erosive findings is a bowel-shaped appearance rounded at the margins. The defects are typically more extensive in width that in depth. BEWE case definitions are as follows: Code 0: no erosion: Code 1: initial loss of surface structures (e. g. shine, perikymata). Code 2: clinically manifest defect, loss of tooth structure over less than 50% of the tooth surface. Code 3: clinically manifest defect, loss of tooth structure over more than 50% of the tooth surface. This estimation of the percentage of the surface affected is based on the most severely affected tooth surface per tooth. The involvement of dentine, which generally occurs from degree two or three, is not named as a graduation criterion.

##### Dental and root caries

To assess dental caries the DMF index is used [24]. This index covers teeth and/or tooth surfaces which are decayed, filled or extracted due to caries. If this assessment is carried out for each tooth surface, adding together the affected tooth surfaces results in the DMFS sum score (S = surfaces). By assessing the findings on tooth level, the DMFT sum score can be calculated (T = teeth). If at least one tooth surface is carious or filled, the whole tooth is classified as a DMF tooth. The D component (D = decayed) stands for tooth or surface destroyed by caries, M (M = missing) for tooth or surfaces extracted due to destruction by caries, and F (F = filled) for a filled tooth or surface due to caries. Coronally, the caries findings are examined visually and not through exploration with a dental probe according to WHO recommendations for epidemiological field studies [25]. This study only uses a blunt periodontal probe to assess sealings or restoration defects. Only clearly diagnosable carious lesions are recorded. With proximal surfaces, the contact with the neighboring tooth frequently makes it difficult to conclusively detect carious lesions. In these cases, the dental investigators are urged to note a defect, where there are typical signs of a proximal lesion shining through. As a general principle, the primary carious surface in each case is recorded as defective. Adjacent areas are also considered as carious if the defect clearly extends to these. Dental restorations are registered in each case where the reason for their presence cannot be be assumed to be other than a carious defect. If both a carious lesion and a dental restoration are present on a tooth surface, the assessment is differentiated on the basis of the degree of severity of the caries. In case of extension of the carious lesion into dentine this surface is classified as carious. In case of initial lesions or carious lesions limited to enamel, however, this is not included in the findings but rather the dental restoration is recorded.

Initial carious lesions are recorded separately, distinguishing between active and inactive lesions. Active initial carious lesions are defined to show a white, rough, and lackluster surface. Inactive initial carious lesions are defined as to present a smooth and glossy surface.

Root caries is examined both as prevalence recording and according to the root caries index (RCI) [26]. A root is assessed as carious if it is possible to establish cavity formation with or without softening. If caries on a root appears to be a continuation of extended crown caries not extending more than 2 mm onto the adjacent root area, no caries finding is noted for the root. In the event of major defects to the root, however, stand-alone root caries is assumed, and this is recorded. For root caries, a distinction is drawn between active and inactive lesions. A brown (yellow, reddish to brown) root surface with varying substance loss and a soft to leathery texture (tactile examination using a blunt probe), usually plaque-covered, is considered as active root caries. Inactive root caries is noted if the substance loss is accompanied by a dark brown to black root surface and hard surface, usually plaque-free. Root surfaces, filled to improve the aesthetic appearance, according to information provided by the subject, are not recorded as filled. Likewise, no dental restoration is recorded if coronal restorations extend up to 2 mm onto roots, as it is assumed that the defect, which was the basis for this restoration, was crown caries. In case of a major restoration to the root, on the other hand, this is recorded as root filling.

In order to be able to calculate the RCI representing the percentage of filled and carious root surfaces relative to the number of exposed root surfaces, healthy but exposed root surfaces are also recorded.

##### Periodontal diseases

The periodontal assessment is performed on the basis of the previous DMS IV, but current developments in epidemiological assessments are taken into consideration [27]. In adults and young olds, all the findings are obtained from the following index teeth [28]: 17, 16, 11, 24, 26, 27, 37, 36, 31, 44, 46, and 47. If there is a missing index tooth, a substitute tooth from the same tooth group is used for the assessment. This means that if 16 and 17 are missing, 18 is used. If 24 is missing, 25 is used, if 11 is missing, 21 is used for assessment. If 21 is also missing, other teeth should be used instead in the following order of priority: 12, 22, 13, 23. If all the substitute teeth from the same tooth group are missing, no evaluation is performed.

In the adult and young olds age cohort, 10% of the subjects are examined using a six-point full-mouth periodontal recording based on a random algorithm process. Because in old olds, it is expected that there will be a reduced natural dentition, a six-point full-mouth periodontal recording is carried out throughout. The 10% subsample approach constitutes a scientific comprise in periodontal epidemiology by determining a so-called inflation factor to correct the periodontal epidemiological underestimation accompanied with the index tooth-specific approach and, on the other hand, to keep the time required for periodontal measurement within acceptable limits [27, 29].

The periodontal pocket probing depth and recession is ascertained using a WHO probe (PCP 11.5B, HuFriedy, Tuttlingen) and is noted with one millimeter increments. The values are up rounded mathematically. The maximum probing pressure is 0.2 N. Making contact with the tooth, the WHO periodontal probe is inserted parallelly to the tooth axis into the sulcus or pocket and the distance from the gingival margin to the sulcus base or pocket base is determined at the following measurement sites per index tooth: mesial-vestibular, medial-vestibular, distal-oral. Entry into the DentaSoft V software is performed to an accuracy of one millimeter.

Gingival recession (resp. hyperplasia) is also determined using the WHO probe and is ascertained at the same sites as the measurement of the periodontal pocket probing depth. The cement-enamel junction (CEJ) serves as a coronal reference point for gingival recession measurement [30]. In the event of a visible CEJ, the distance between CEJ and the gingival margin is measured to an accuracy of one millimeter as a positive value. If the gingival margin is positioned coronally to the CEJ, it is detected by using the probe tilted outwards by approximately 45° and carefully moving the probe in an upward and downward direction and it is noted with a minus value. If the CEJ is not discernible due to a dental restoration or a crown, then it should be determined arbitrarily on the basis of the anatomy of the neighboring teeth. If it cannot be determined due to extensive prosthetic provision, the gingival recession (resp. hyperplasia) cannot be documented. Attachment loss is calculated as the sum of periodontal pocket probing depth and gingival recession.

##### Gingivitis

In children, the papilla bleeding index (PBI) is determined instead of the above described periodontal measurement as advanced periodontal disease is not expected in this age cohort [31]. Papilla bleeding is provoked using a WHO probe by gentle probing the sulcus of the mesial and distal papilla. The stroking pressure is at maximum of 0.2 N. One-off gentle probing from the papilla base up to the papilla tip is performed. The probing begins on the distal-vestibular site on tooth 16 and is continued until the mesial-vestibular site on tooth 11. Subsequently, the extent of any bleeding is immediately assessed. This is followed by measurement and evaluation in the second quadrant orally. A similar approach is taken from the vestibular side in the third quadrant and again orally in the fourth quadrant. The PBI scale is as follows: Code 0: no bleeding. Code 1: appearance of one bleeding point. Code 2: appearance of different isolated bleeding points on less than half of the coated length. Code 3: the interdental triangle fills with blood shortly after probing. Code 4: severe bleeding from the papilla region.

##### Prosthodontics

Most prosthetic findings, such as crowns or bridge works, emerge from the tooth-specific findings at the beginning of the clinical examination. At this point, the type of denture is registered. The type of denture, separately for upper and lower jaw, is recorded as follows: resin partial denture with curved retention elements, model cast denture, combined denture with complex anchorage (telescopic, bar, attachment denture, hybrid denture excl. anchorage element on root caps), full denture. For each denture in the upper and lower jaw, information is recorded about the wearing behavior. Sporadic wearing (to look better in company or similar) is rated as non-wearing.

##### Index of oral functional capability

A four-level index of oral functional capability (IOFC) is measured in old olds. The determination of IOFC is a dental investigator’s estimation [32]. The IOFC consists of three dimensions: treatment potential level, oral hygiene ability, and personal responsibility.

The treatment potential level refers to whether dental treatment may performed consistent with treating healthy subjects, or if there should be certain limitations be expected due to decreased capability (for example number and length of appointments, diagnostic options, medical risk factors, medication, type of dental treatment concept). Neither the financial situation nor the dental status of the subject has any impact on the determination of the treatment potential level. When assessing the oral hygiene ability, the question must be answered whether the subject can participate in an individual prophylactic dental treatment and whether the subject has the motor function and cognitive skills to understand the instructions on oral hygiene and implement these in her/his daily oral and denture hygiene regime. Personal responsibility refers to the subject ability to decide on the one hand to seek dental services and on the other hand to individually organize this visit. The index of oral functional capability is calculated in a four point capability level scale: normal, slightly reduced, considerably reduced, and none.

### Participant timeline

The time calculation is essentially determined by the clinical assessments. It is calculated per subject as follows: children: ten minutes, adults: 25 minutes (additional ten minutes in case of 10% periodontal subsample), young olds: 25 minutes (additional ten minutes in case of 10% periodontal subsample), and old olds: 40 minutes.

Completion of the socio-scientific questionnaire is calculated with ten minutes for children, 15-20 minutes for adults and young olds, and 10-15 minutes for old olds. Alternatively in old olds, a confidential person may complete the questionnaire if needed.

### Data collection methods and management

As soon as the contact person arrives at the study sample point, he has exclusive control over the data entry. In the week of study examinations and surveys, on the other hand, only the interviewer and, as appropriate, the dental investigator, is able to input data in the address log. The address and assessment data are entered separately in software programs, specially designed for the study. The interviewer receives a copy of the address data for the particular study sample point and passes the ID of the subject to the dentist for clinical examination. The address database contains the subject’s address, the contact attempts, appointments, response codes, presence of completed questionnaires, willingness to be questioned again, presence of informed consent. Every day, the address and assessment data is sent in encrypted form to the operation center. The data is stored on non-public data storages. Address and assessment data are kept separately. During the investigation week, the completed questionnaires and informed consents are stored in lockable cases. After a sample point has been completed, the interviewer sends the completed questionnaires and informed consents in separate envelopes to the operation center.

### Quality control

At all times, it is ensured that personal address and content-related data (questionnaire) as well as assessment data are kept strictly separate from each other. The data is matched by means of the subject ID. The returned questionnaires are paginated and recorded in a responses database. After the data has been scanned in, a checking and verification process is performed. In close consultation with the principal investigator, plausibility check rules are determined for this purpose (permitted answers, value ranges, filter categories, questions depending on each other etc.). Before scanning in of the incoming questionnaires takes place, quality control is performed with respect to completeness.

### Statistical methods

The operation center is responsible for the statistical analysis of the data according to good epidemiological practice [33]. Before starting statistical analysis, a design loading is conducted to eliminate the disproportional sampling procedure on West/East Germany level. The survey features are illustrated descriptively by means of suitable tabulation. To illustrate distributions, bar charts displaying mean, min. and max. values are used. Calculations of several indices are performed on basis of the recorded data: attachment loss (AL), basic erosive wear examination (BEWE), bleeding on probing (BOP), community periodontal index (CPI), oral functional capability (IOFC), MFS/T index, molar incisor hypomineralization (MIH), papilla bleeding index (PBI), root caries index (RCI), and sense of coherence total score (SOC). To illustrate the differences between different groups, relative risks (RR) are calculated; these are well suited to segmentation analyses. In order to highlight links between different criteria, correlation analyses are carried out. Results are checked for statistical significance. Calculation of the socio-economic status (SES) is based on a model of three variables (school level, occupation position, household income). Using an estimation model, a non-response analysis is performed to investigate whether there are differences between the participants and non-participants of the study. To compare the results with previous DMS studies, the calculated values and indices are finally compared with the earlier data. Statistical analysis is calculated according to a detailed statistical analysis plan (SAP).

### Informed consent materials and biological specimens

As this is a socio-epidemiological study, a clinical assessment of the oral cavity is carried out in addition to a written survey. The clinical examination approximately corresponds to the scope covered by a dental check-up examination. The assessment is performed non-invasively and no biological material is taken from the subject.

Participation in the study requires a written informed consent from the selected participants. Without an informed consent, participation in the Fifth German Oral Health Study is not possible. All subjects receive information about the purpose of the study and a declaration on data protection. The information is provided by the interviewer at the investigation center before the start of the assessment. In addition, the subject has to sign the informed consent for participation in the study at this point. For the 12-year-olds the signature of parents or legal guardian is required. If people (possibly in advanced years) are cared for by a legal guardian, then the latter’s signature must be obtained.

## Discussion

The DMS studies introduced in 1989 are the only sociological and clinical examinational oral health studies conducted in Germany on a national population-based representative level. Updated prevalence and trend analyses of key oral diseases are, therefore, of major epidemiological and health services research interest. For global comparison, dental caries trends in the young population, represented by 12-year-olds, are the center of attention and likewise a monitoring instrument of the success of preventive measures. Caries experience in this age cohort has been declining since decades, and children from previous DMS studies reach adult age by now. In this respect, DMS V caries prevalence in adults is of further interest from a public health point of view, as a sustainable caries preventive effect from childhood to adulthood would be plausible to expect. Another focus of interest is the periodontal disease burden, especially in old age cohorts. To identify periodontitis prevalence, the epidemiological methods in DMS V were improved according to current recommendations [27]. Instead of using specific (Ramfjord) index teeth for periodontal measurements, a six-sites per tooth full-mouth recording is performed in randomly selected every tenth subject to calculate a sample-wide inflation factor. For the first time, the age cohort between 75 and 100 years of age is included in a DMS study. Enormous demographic changes are in progress in Germany and they are induced by the double effect of an ageing population – on the one hand, people grow older, on the other hand, the portion of young olds and old olds in the total population is continuously increasing because of a declining birth rate. Therefore, oral health epidemiological data of this population will be fundamental for oral health care planning. Finally, DMS V will present findings of some oral diseases like molar incisor hypomineralization that have not been investigated on a representative population-based level in Germany yet.

It is the strength of a single cross-sectional study to describe disease prevalence as far as a robust study design features a representative participant enrollment. Since the cross-sectional DMS studies are repeated for the fourth time they can be used for trend analyses to monitor disease trends and to monitor the change of risk factors. So performed with several DMS study data demonstrating trends in dental health of adults in West and East Germany after reunification [34], or identifying changes in determinants of functional health in adults in DMS studies between 1989 and 2005 [9]. As limitation of those secondary analyses it must be stated that de facto life span conclusions might be confounded due to several reasons. One reason is to be seen in the development of dental health driven by medical and societal progress. At intervals of about 20 years, different so-called dental generations can be identified with different treatment concepts [35]. In Germany, they might be identified as follows: birth cohorts before 1950: extraction and dentures generation; 1950s and 1960s: filling generation; 1970s and 1980s: fluoride generation; 1990s and younger: future generation. From this socio-medical point of view, even trend analyses between these dental generations appear difficult. With DMS V, it will be the first time that trend analyses on basis of specific birth cohorts may be performed as the birth cohorts of adults correspond to the birth cohorts of adolescents in DMS I and DMS II, the birth cohorts of young olds correspond to the birth cohorts of senior adults in DMS II, and the birth cohorts of old olds correspond to the birth cohorts of young olds in DMS II and DMS IV.

International comparisons on oral diseases can be drawn from WHO oral health databanks. The WHO Collaborating Centre for Education, Training and Research in Oral Health at Malmö University, Sweden, established a standardized reporting system, the Country/Area Profile Project (CAPP) for key oral diseases [36]. Epidemiological comparisons demonstrate that dental caries in children in Germany is top ranking among those countries with the lowest caries experience in this age cohort [4]. For periodontal diseases, periodontal profiles are available for global comparison, too. Based on the precursor DMS IV study, it must be acknowledged that periodontitis is highly prevalent in Germany [34]. The current DMS V will therefore not only actualize the oral health epidemiological data records in Germany but also contribute CAPP for up to date scientific comparisons.

On the basis of WHO global goals for oral health 2020 [37], also ambitious goals for oral health in Germany 2020 were presented [38]: In 12-year-olds, mean DMFT index should be below 1.0 teeth. As in precursor DMS IV in 2005, mean DMFT in children already scored 0.7 [4], so probably this oral health goal will be attained. In 35- to 44-year-olds, mean M component of the DMFT index (MT) should not exceed 2.0 teeth. Further, severe periodontal diseases should not exceed 20%. In DMS IV, mean MT was 2.7 in adults [4], and severe periodontal diseases reached up to 8% [34]. Finally, oral health goals for 65- to 74-year-olds were defined with a maximum of 20% severe periodontal diseases, and less than 15% of the respective population being edentulous. Severe periodontal diseases in DMS IV in young olds rised up to 22%, and 22.6% were edentulous in DMS IV [34]. The DMS V study will be the last crucial reference point to benchmark these goals to oral health in Germany.

### Ethical approval

The Fifth German Oral Health Study (DMS V) has been approved by the Institutional Review Board (IRB) of the North Rhine medical association, Duesseldorf (DMS V registration number 2013384). This study is registered at the German Health Services Research Data Bank (DMS V registration number VfD_DMSV_13_002152).

## Abbreviations

BEWE: Basic erosive wear examination
BOP: Bleeding on probing
CAPP: Country/area profile project
CEJ: Cement-enamel junction
IRB: Institutional review board
ISO: International Standard Organization
DMFS: Decayed, missing, filled surfaces
DMFT: Decayed, missing, filled teeth
DMS (I-IV): German oral health studies (I-IV)
DMS V: Fifth German Oral Health Study
IOFC: Index of oral functional capacity
IDZ: Institute of German Dentists
MIH: Molar incisor hypomineralization
PBI: Papilla bleeding index
PSR: Periodontal screening and recording
RCI: Root caries index
RR: Relative risk
SAP: Standard analysis plan
SES: Socio-economic status
SOC: Sense of coherence
WHO: World Health Organization.
